# Insights into Chemical Bonds for Eliminating the Depletion Region and Accelerating the Photo-Induced Charge Efficient Separation toward Ultrasensitive Photoelectrochemical Sensing

**DOI:** 10.3390/bios13110984

**Published:** 2023-11-13

**Authors:** Shuai Wang, Haihan Yu, Shenguang Ge, Yanhu Wang, Chaomin Gao, Jinghua Yu

**Affiliations:** 1 School of Chemistry and Chemical Engineering, University of Jinan, Jinan 250022, China; 2 Institute for Advanced Interdisciplinary Research, University of Jinan, Jinan 250022, China; 3 Key Laboratory for Applied Technology of Sophisticated Analytical Instruments of Shandong Province, Shandong Analysis and Test Center, Qilu University of Technology (Shandong Academy of Sciences), Jinan 250014, China

**Keywords:** photoelectrochemical sensor, chemical bond, charge separation

## Abstract

The empty-space-induced depletion region in photoelectrodes severely exacerbates the recombination of electron–hole pairs, thereby reducing the photoelectrochemical (PEC) analytical performance. Herein, the chemical bond that can suppress the potential barrier and overcome the high energy barrier of out-of-plane Ohmic or Schottky contact is introduced into the PEC sensor to eliminate the depletion region and dramatically promote the separation of electron–hole pairs. Specifically, three-dimensional (3D) hierarchically wheatear-like TiO_2_ (HWT) nanostructures featuring a large surface area to absorb incident light are crafted as the substrate. The facile carbonized strategy is further employed to engineer the Ti-C chemical bond, serving as the touchstone. The average PL lifetime of HWT-C (4.14 ns) is much shorter than that of the 3D HWT (8.57 ns) due to the promoting effect of the chemically bonded structure on carrier separation. Consequently, the 3D HWT-C covalent photoelectrode (600 μA/cm^2^) exhibits a 3.6-fold increase in photocurrent density compared with the 3D HWT (167 μA/cm^2^). Ultimately, the model analyte of the tumor marker is detected, and the linear range is 0.02 ng/mL–100 ng/mL with a detection limitation of 0.007 ng/mL. This work provides a basic understanding of chemical bonds in tuning charge separation and insights on strategies for designing high-performance PEC sensors.

## 1. Introduction

**Scheme 1:**
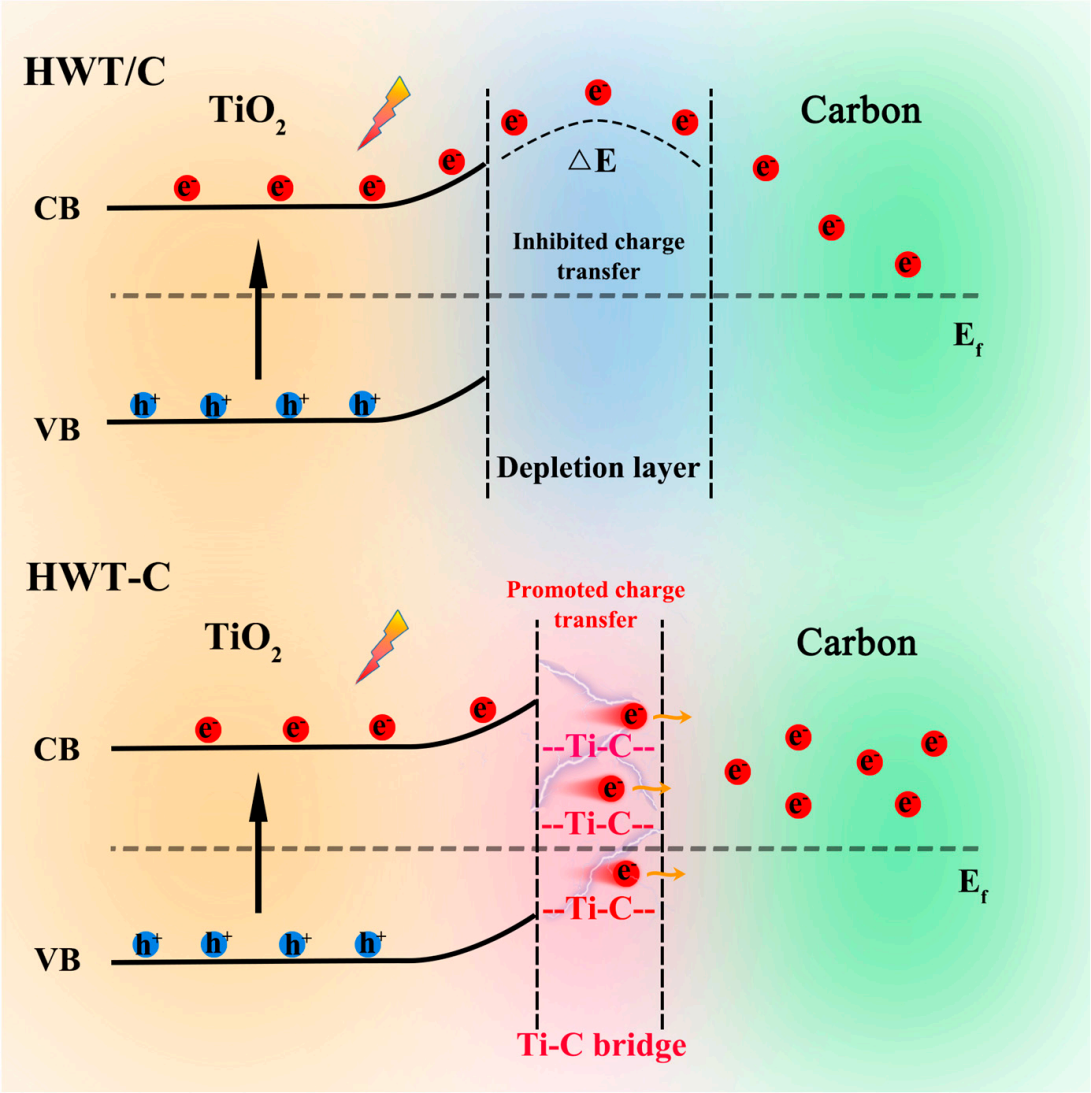
The charge transfers in HWT-C with a chemically bonded interface and in HWT/C without a chemically bonded interface.

Emerging as a burgeoning analytical technique, photoelectrochemical (PEC) bioanalysis has been extensively investigated in recent years on account of its merits of high sensitivity, low background signals, and simple instrumentation [1,2,3]. Essentially, in PEC bioanalysis, the analytes are detected through monitoring the variations of the obtained photocurrent density signals generated by the photoactive materials [4,5]. Therefore, the analytical performance of PEC bioanalysis strongly relies on the obtained photocurrent intensity. That is to say, the carrier separation efficiency of photoactive material, as one of the crucial steps determining the intensity of photocurrent, plays a decisive role in analytical performance of PEC bioanalysis [6,7,8]. In this circumstance, enhancing the sensitivity hinges on boosting the efficient separation of photo-induced electron–hole pairs and suppressing their recombination. To pursue a highly effective approach that can achieve the above goal appears to be a major concern. Up to date, strategies such as establishing a built-in electric field and forming heterojunctions have been explored [9,10,11,12]. For example, hollow CdIn_2_S_4_/In_2_S_3_ heterostructured microspheres are prepared, and a PEC sytosensor for HepG2 cells for ultrasensitive sensing via a specific recognition of the CD133 protein on the cell surface with the respective aptamer is constructed based on that heterostructure [13]. Tang et al. report a tunable signal-on PEC biosensor for the detection of cardiac troponin I based on a Z-scheme heterostructure substrate of water-stable Zr metal-organic framework-modified TiO_2_ nanorods [14]. A WO_3_/CdS heterojunction photoanode is engineered, and its potential in the PEC determination of Cu^2+^ in an aqueous solution has also been explored [15]. Additionally, we have also demonstrated that the introduction of a ferroelectric polar charge-created electric field and bidirectional modulation of the photoinduced charge carrier separation strategy can effectively facilitate the separation of electron–hole pairs [16,17]. Although distinguished achievements have been made, some remaining challenges still need to be resolved. For example, the construction of heterojunction photoelectrodes has proven to be a powerful method to facilitate the separation of electrons and holes, thereby realizing the admirable photocurrent and analytical performance [15,18,19]. Nevertheless, the random accumulation of two different semiconductors through compound manipulation in composite materials-based heterojunction photoelectrodes may lead to the obvious empty spaces forming as the depletion region and recombination centers to further impede the transfer of electrons to a large extent [20]. In that case, additional energy is needed for the electrons to cross this depletion layer, as shown in Scheme 1. Moreover, as opposed to a single photoactive material, the transport of photo-induced electrons and holes between composite materials with different band gaps must overcome the high energy barrier of out-of-plane Ohmic or Schottky contact during an intramolecular cascade. Such a depletion layer in a photoelectrode is undesirable owing to its hindering effect on carrier transport, thereby leading to a low photocurrent response and unsatisfactory PEC analytical performance. Therefore, elucidating the side effect mechanism of the depletion layer and exploring other effective strategies for eliminating the depletion region and, hence, enhancing the PEC analytical performance are anticipated.

Noticeably, the interfacial parameters between the heterojunction structure—for example, contact area, band bending, and chemical bond—all play a vital role in facilitating charge separation [21]. Intriguingly, the chemically bonded structure based on a sufficient chemical connection serving as an atomic-level charge transport channel can efficiently eliminate the depletion layer, shorten the charge transfer path, and reduce the transportation resistance of charge carriers [22,23,24], which can be treated as a feasible way to resolve the above problem in a PEC sensor. Additionally, the chemically bonded structure can also efficiently suppress the potential barrier (ΔE) and facilitate the vectorial transfer of carriers at the interface, thereby achieving the separation and transport of electron–hole pairs [22]. The carrier transport in chemically bonded photoelectrode does not need to overcome the high energy barrier of out-of-plane Ohmic or Schottky contact compared with composite materials-based photoelectrodes. Moreover, the photoelectrode with the chemical bond is more stable, owing to the strong chemical connection [25,26]. Most importantly, in the field of photocatalysis, the promoting effect of chemical bonds on the efficient separation of charge carriers and ultimately achieving improved photocatalytic performance has been widely investigated. For example, the α-Fe_2_O_3_/Bi_4_MO_8_Cl dot-on-plate Z-scheme junction with a strong internal electric field endowed by the chemical bond has been prepared by the Yu group, achieving sensitive and selective photo-oxidation of aromatic alcohols [27]. Yu et al. report that by decorating the Co_2_P cocatalyst onto the surface of black phosphorus nanosheets, they can construct Co-P chemical bonds. Based on the results of the first-principle calculation and photocurrent measurement, the Co-P chemical bond serves as the atomic-level chargeflow steering, which can facilitate the separation of photogenerated charge carrier transfer between BP and Co_2_P, thereby enhancing the photocatalytic performance of the Co_2_P/BP photocatalyst for H_2_ generation [28]. Furthermore, the TiO_2_/g-C_3_N_4_ composite with a strongly coupling (Ti)_2_-N-C bond at the interface was synthesized using a simple hydrothermal method, and the separation and transfer of the photogenerated carriers at the hybrid interface of the composites have been realized according to the results of transmission electron microscopy, X-ray photoelectron spectroscopy, and nuclear magnetic resonance [29]. Hence, introducing a chemical bond into the photoelectrode in a PEC sensor can enable the spatial separation of the electron–hole pairs and enhance the analytical performance. However, despite such attractive properties, this promising strategy is seldom employed in the field of PEC bioanalysis.

Titanium dioxide (TiO_2_) has been widely studied in PEC sensors due to its strong optical absorption, outstanding chemical stability, and low cost. However, its large bandgap width and fast carrier recombination seriously affect its further application [30]. Zhou et al. synthesize an ultrasensitive TiO_2_/MXene/CdS QDs heterostructure as a PEC immunosensor, favoring energy level matching and fast electron transfer from CdS to TiO_2_ with the help of an ultrathin MXene nanosheet [31]. A novel PEC sensor for cholesterol detection based on titanium dioxide inverse opal and CH_3_NH_3_PbBr_3_ quantum dot heterojunction on ITO glass has also been constructed [32]. In addition, Qu et al. developed a simple but low-cost PEC sensor for the highly sensitive sensing of Staphylococcus aureus based on Cu-C_3_N_4_-TiO_2_ heterostructures as photoactive materials [33]. Moreover, carbon (C) has attracted much interest owing to its great biocompatibility and abundance of natural raw materials. At the same time, C is also widely used for combining with semiconductors to improve its photoelectric performance because of its fast charge transfer and strong electronic storage capacity [34]. In this work, three-dimensional (3D) hierarchically wheatear-like TiO_2_ (denoted as HWT) arrays featuring biocompatibility and low cost [35,36,37,38], were used as the backbone semiconductor, and a 3D HWT-C core–shell photoelectrode with the Ti-C chemically bonded interface was also successfully prepared. Based on this, an ultrasensitive PEC sensor for the detection of prostate-specific antigen (PSA) is constructed, and obviously enhanced photocurrent signals are achieved, thereby dramatically enhancing the sensitivity. Specifically, as the concentration of PSA increases, the steric hindrance effect continues to strengthen, leading to a decrease in photocurrent and achieving highly sensing PSA.

## 2. Materials and Methods

### 2.1. Chemicals and Characterizations

Bovine serum albumin (BSA), tetrabutyl titanate, and ascorbic acid (AA, ≥99.0%) were obtained from Sigma-Aldrich Chemical Co. (St. Louis, MO, USA) PSA and antibodies for PSA were obtained from Shanghai Linc-Bio Science Co. LTD. (Shanghai, China). Chitosan, glutaraldehyde, sodium hydroxide (NaOH), ethanol (CH_3_CH_2_OH), hydroquinone, TiCl_3_, and hydrochloric acid (HCl) were purchased from Aladdin (Shanghai, China). All reagents were used as received with an analytical grade. Ultrapure water was used in all experiments and was obtained from a Lichun water purification system (Jinan, China) (>18.25 MΩ cm). A PLS-SXE 300 xenon lamp (Beijing Changtuo Co. Ltd. (Beijing, China)) equipped with a monochromator was used as the irradiation source. The morphologies of the samples were characterized using a QUANTA FEG 250 thermal field emission scanning electron microscopy (FEI Co., Hillsboro, OR, USA). The photocurrent response was measured with a CHI 660C electrochemical working station (Shanghai Chenhua Instrument Co., Shanghai, China) with a three-electrode system: an HWT-C photoelectrode as the working electrode, a Pt wire as the counter electrode, and a saturated Ag/AgCl electrode as the reference electrode, respectively. The wavelength of light used to obtain the photocurrent response is 450 nm. The phase characterization was performed via X-ray diffraction (XRD) using a D8 advance diffractometer system equipped with Cu Kα radiation (Bruker Co., Berlin, Germany). The UV-visible (UV-vis) optical absorption spectrum was measured with a Shimadzu UV-3600 spectrophotometer. Steady-state photoluminescence (PL) and time-resolved PL measurements were acquired using an FLS980 fluorescence spectrometer (Edinburgh Instruments, Livingston, UK). The XPS results were obtained using X-ray photoelectron spectroscopy (ESCA210, VG) with an Al Kα X-ray generator (1486.6 eV).

### 2.2. Synthesis of 3D HWT and HWT-C Arrays

In order to obtain 3D HWT arrays, the TiO_2_ NRs were first prepared accordingly to our previous report [38]. In brief, 30 mL of ultrapure water was added to 0.2 mL of HCl (12 M), followed by adding a 0.2 mL TiCl_3_ solution to prepare the branch-forming solution. Then, the obtained TiO_2_ NRs/FTO sample was placed at an angle against the wall of the breaker with the conductor facing up. The sealed breaker was heated for 1 h in an oven under at 80 °C. Finally, the resulting sample was rinsed thoroughly with ultrapure water and allowed to dry at 60 °C.

Then, the 3D HWT-C sample was prepared as follows: 0.4 g of hydroquinone was added to ethanol (40 mL) while stirring. Then, the as-obtained HWT array sample was rinsed into the solution under high-purity nitrogen gas and stirred for 4 h, followed by washing with ethanol and ultrapure water. Finally, the resulting sample was carbonized at 500 °C for 6 h under an argon atmosphere with a heating rate of 5 °C min^−1^. Furthermore, in order to emphasize the role of the Ti-C chemical bond, a sample of 3D HWT/C without a chemical bond was also prepared.

### 2.3. Fabrication of the PEC Sensor

Firstly, the HWT-C photoelectrode was activated with chitosan and glutaraldehyde. Briefly, 20 μL of chitosan solution (0.1 wt%) was added onto the photoelectrode and then washed with 0.1 M NaOH and ultrapure water. After that, 25 μL of glutaraldehyde solution (5.0 wt%) was added to the surface of the photoelectrode and kept for 60 min, followed by washing thoroughly. Then, 30 μL of PSA antibody solution was spread onto the activated photoelectrode and incubated at 4 °C for 6 h, then washed thoroughly. Consequently, 30 μL of blocking buffer (0.25% BSA dissolved with 0.1 M of phosphate-buffered saline (PBS, pH 7.4)) was used to block the non-specific binding sites of the photoelectrode at 4 °C for 2 h.

### 2.4. Assay Procedure of the PEC Sensor

The PSA antigen solution with different concentrations was dropped onto the photoelectrode and incubated at 37 °C for 1 h, followed by washing thoroughly with ultrapure water. The photocurrent intensity of the PEC sensor was measured with a CHI 660C electrochemical working station (Shanghai Chenhua Instrument Co., Shanghai, China) in a PBS solution (0.1 M, pH 7.4) containing 0.1 M ascorbic acid, illuminated by 450 nm of light. A PLS-SXE 300 xenon lamp (100 mW cm^−2^) was utilized as the irradiation source, which was switched on and off every 20 s, and the applied potential was 0 V.

## 3. Results and Discussions

### 3.1. Characterization of HWT and HWT-C Samples

**Figure 1:**
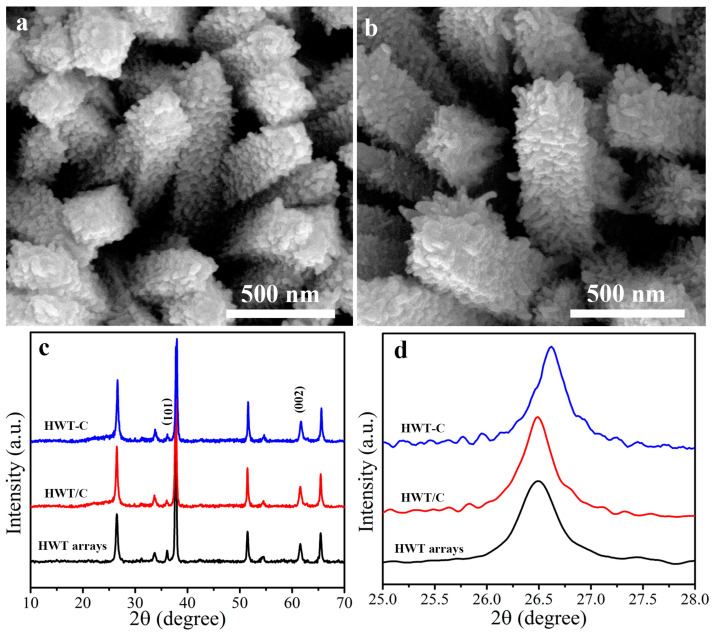
SEM images of (**a**) 3D HWT array and (**b**) HWT-C; (**c**) XRD pattern of 3D HWT array, HWT@C, and HWT/C samples; (**d**) corresponding local enlargement of the 2θ diffraction range.

The morphologies of the obtained samples were characterized with scanning electron microscopy (SEM) images. As illustrated in Figure 1a, the quasi-vertically standing of 3D HWT arrays with a diameter of around 250 nm onto the FTO substrate was observed. Furthermore, the growth mechanism of the 3D HWT has also been proposed based on time-dependent growth processes (Figure S1). After carbonization, the surfaces became rough, but the 3D geometry structure of HWT was still maintained (Figure 1b). In order to validate the crystallinity and the successful formation of the chemical bond, X-ray diffraction (XRD) measurements were carried out, as shown in Figure 1c. The diffraction peaks of 3D HWT arrays, 3D HWT-C, and 3D HWT/C samples were well indexed to the rutile TiO_2_ phase (JCPDS no. 12-7216) [39,40]. Importantly, the enlarged profiles in Figure 1d exhibited that, compared with pristine 3D HWT arrays and 3D HWT/C, the (101) diffraction peaks in HWT-C slightly shifted to a higher degree, attributing to the shrink during carbonization and the stress induced by Ti-C bonds [41], suggesting the formation of a Ti-C chemical bond.

**Figure 2:**
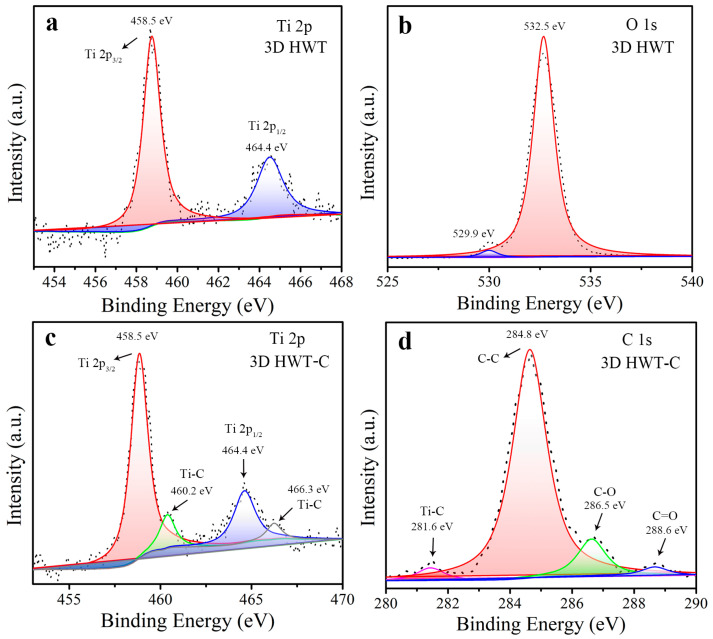
XPS of 3D HWT arrays and HWT-C samples. (**a**) Ti 2p and (**b**) O 1s spectra of HWT arrays. (**c**) Ti 2p and (**d**) C 1s spectra of HWT-C. The dot curves are experimental data.

Moreover, X-ray photoelectron spectroscopy (XPS) was also performed to further determine the successful formation of the Ti-C chemical bond and the composition of the samples, as shown in Figure 2. The full-range XPS spectra of HWT-C indicated that the main elements of the samples were Ti, O, and C (Figure S2). High-resolution XPS spectra of 3D HWT arrays exhibited Ti 2p and O 1s electron peak regions, as shown in Figure 2a,b. The two peaks centered at 458.5 and 464.4 eV were attributed to Ti 2p_3/2_ and Ti 2p_1/2_, respectively (Figure 2a) [42]. In addition, the two peaks at 532.5 and 529.9 eV in the O 1s spectrum (Figure 2b) were ascribed to the lattice oxygen and surface hydroxyl groups, respectively [43]. As shown in Figure 2c, compared with pristine 3D HWT arrays, two additional peaks centered at 460.2 and 466.3 eV of HWT-C were also observed, indicating that the Ti-C chemical bond was formed between titanium and C [44]. Furthermore, the successful formation of the chemical bond was also confirmed with the C 1s spectra, as shown in Figure 2d. The binding energy of 282.8 eV was assigned to sp^2^ hybridized C, and another two peaks located at the binding energies of 286.5 and 288.6 eV were attributed to C-O and C=O bonds, respectively [45]. Moreover, a peak centered at 281.6 eV was observed, further implying the successful formation of a Ti-C chemical bond between the interfaces [46]. The content of C on the surface of HWT-C was about 79.6%, which was quantified with the high-resolution XPS spectra, confirming the successful preparation of the HWT-C sample.

### 3.2. Investigation on the Promotion of Chemical Bonds on Carrier’s Separation

**Figure 3:**
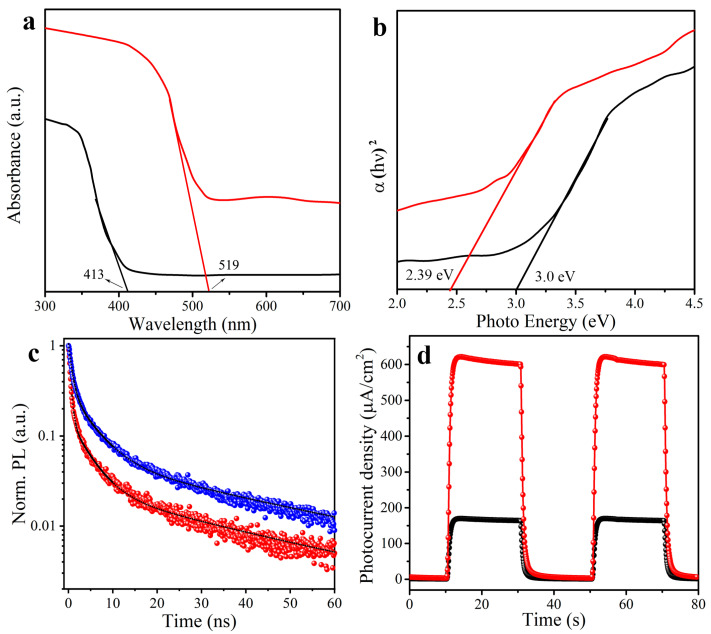
(**a**) UV-Vis diffuse reflectance spectra of the HWT array (black curve) and HWT-C samples (red curve). (**b**) Plot of (αhν)^2^ vs. energy (hν) of HWT array (black curve) and HWT-C samples (red curve). (**c**) TRPL spectra of HWT (blue curve) and HWT-C (red curve) samples. (**d**) Photocurrent plots of HWT array (black curve) and HWT-C (red curve) samples.

The energy band structure of 3D HWT arrays and HWT-C was also investigated in order to gain deeper insight into the promoting effect of chemical bonds on carrier charge separation. Figure 3a shows the UV-Vis diffuse reflectance spectra (DRS) of 3D HWT arrays and HWT-C samples. As shown, the HWT arrays exhibited an absorption edge of 413 nm. The HWT-C showed broadened light absorption both in the UV and visible-light regions, with an absorption edge of 519 nm due to the formed Ti-C chemical bond, which was coincident with previous reports [47]. As shown in Figure 3b, the energy band gap (E_g_) could be obtained by extrapolating the linear part of the (αhν)^2^ curve vs. photon energy (hν), which was obtained through the transformation of the UV-Vis DRS plot using the Kubelka–Munk function. The estimated band gaps of 3D HWT arrays and HWT-C were characterized as 3.0 and 2.39 eV, respectively.

To further evaluate the facilitation of the Ti-C chemical bond on the separation efficiency of photo-induced electrons and holes, steady-state photoluminescence (PL) measurement, which was formed via the recombination of electron–hole pairs, was performed [48,49]. As shown in Figure S3, an obvious PL emission peak was found for pristine 3D HWT arrays, attributing to their poor separation of charge carriers. Notably, the PL emission of HWT-C significantly decreased, implying that the Ti-C chemical bond could effectively facilitate the separation of charge carriers. Furthermore, to gain further insight into the charge separation dynamics of samples, time-resolved PL (TRPL) decay was also conducted (Figure 3c). The biexponential model was used to fit the date, and the obtained parameters are provided in Table S1. As shown in Table S1, after the formation of the Ti-C chemical bond, the fast decay component of *τ*_1_ that was related to charge transfer enhanced obviously, on the contrary to the trend of *τ*_2_, which was the slow decay component related to radiative recombination [50,51,52]. The average PL lifetime (*τ*_ave_) was calculated using the formula of $\tau_{ave}=\frac{\sum_{i=0}^{n}A_{i}\tau_{i}^{2}}{\sum_{i=0}^{n}A_{i}\tau_{i}}$ and revealed that the *τ*_ave_ of the HWT-C (4.14 ns) sample was much shorter than that of 3D HWT arrays (8.57 ns), which was in accordance with the quenching trend of steady-state PL in Figure S3. That indicated that the existence of a chemical bond could effectively extract charge carriers from TiO_2_. Such a conclusion was also supported by the photocurrent response, as shown in Figure 3d. The observed photocurrent response in the shape of nearly vertical rising and falling implied the quick charge transport in the samples. It can be seen that, compared with 3D HWT arrays, a remarkably increased photocurrent density was observed for the HWT-C, demonstrating the presence of a Ti-C chemical bond could significantly decrease electron and hole recombination, agreeing with the results of PL and TRPL.

### 3.3. Feasibility of the PEC Sensor

**Figure 4:**
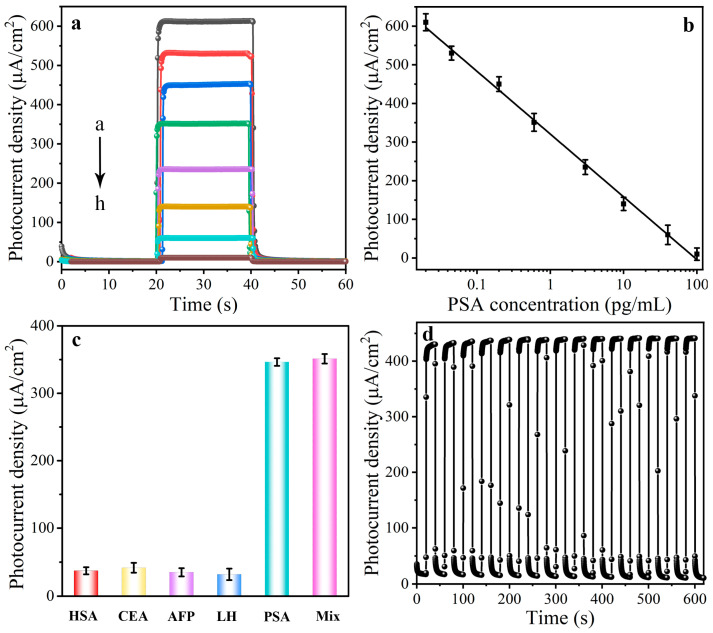
(**a**) Photocurrent response of the PEC sensor with variable PSA concentrations (a to h: 0.02, 0.045, 0.2, 0.6, 3, 10, 40, and 100 ng/mL). (**b**) The calibration plots of the PEC sensor versus the logarithm of the PSA concentration. (**c**) Selectivity of the PEC sensor for PSA detection over interferences. (**d**) Stability of the PEC sensor with PSA concentrations of 0.2 ng/mL.

Finally, the PEC sensing performance of the as-obtained photoelectrode was investigated. The photocurrent response of the photoelectrode after being incubated with various concentrations of PSA was verified. As shown in Figure 4a, with the concentration of PSA raised, more and more conjugates were captured on the surface of the photoelectrode, resulting in a gradual decrease in the photocurrent response owing to the steric effect. The linear regression equation was calculated as I = 320.7 − 162.4 log*c* with a correlation coefficient of 0.996. The photocurrent intensity was related to the concentration of PSA, with a range of 0.02 pg/mL to 100 ng/mL, and the detection limit (LOD) was 0.007 pg/mL. Such a larger linear range and low LOD were attributed to the Ti-C chemical bind. Furthermore, the as-constructed PEC sensor exhibited acceptable analytical performance (Table S2).

### 3.4. Optimization of the Detection Conditions

In addition, the detection conditions of the PEC sensor were also optimized, as shown in Figures S4 and S5. The value of pH played an important role in the analytical performance of as-established PEC sensor, and its effect is shown in Figure S4. The photocurrent response rose with the increase in pH and reached its maximum value at 7.4, which was selected for further experiments. In addition, the effect of incubation time on the photocurrent response with a PSA concentration of 10 ng/mL was also assessed, as shown in Figure S5. The photocurrent decreased along with the increase in incubation time in the range of 0 to 40 min and reached a constant state after 40 min. Thus, 40 min was selected as the optimal incubation time.

### 3.5. Selectivity, Reproducibility, and Stability

The selectivity of the established PEC sensor was investigated with 10 ng/mL of CEA, alpha fetal protein (AFP), luteotropic hormone (LH), and human serum albumin (HSA) serving as interfering agents. The results in Figure 4c indicated that only the PSA could cause the obvious response change, and no significant photocurrent change was observed for all these interfering agents. Furthermore, interassay precision between 10 devices was involved to assess reproducibility. The RSDs for parallel detection of 0.1, 1, and 10 ng/mL PSA were 3.89%, 3.12%, and 3.46%, respectively, implying satisfactory reproducibility. Moreover, the time-based photocurrent response of the PEC sensor with the light on and off for 500 s was performed to study the stability property (Figure 4d). As seen, the almost unchanged photocurrent intensity suggests the great stability of the as-constructed sensor. Moreover, the feasibility of an as-constructed sensor for real sample detection was also investigated with a standard addition method (Table S3). The recovery was from 96% to 105%, suggesting the great application potential of the PEC sensor. As described in Table S4, the PEC sensor has a wider linear detection range and a lower LOD compared to other materials, indicating the promoting effect of Ti-C bonds on carrier separation and hence enhancing PEC analytic performance.

## 4. Discussion

In this work, the chemical bond concept was first introduced into the photoelectrode of PEC bioanalysis to eliminate the depletion region between photoactive materials and promote the separation of electron–hole pairs, thereby enhancing the analytical performance. Firstly, the 3D HWT arrays were prepared with the hydrothermal method as the substrate, and the facile carbonized strategy was further employed to obtain the Ti-C chemical bond. Profiting from such robust chemically bonded interface contacts, effective spatial separation of electron–hole pairs and prominent photocurrent density were achieved, which were evidenced by PL, TRPL, and photocurrent response characterizations. Based on the larger obtained photocurrent signal, the ultrasensitive detection of PSA was realized. Exploiting the concept of the chemical bond beyond the existing strategy will offer new insights into understanding the role of the chemical bond in tuning charge separation and designing high-performance PEC bioanalysis.

## Data Availability

Data are contained within the article.

## Supplementary Materials

The following supporting information can be downloaded at https://www.mdpi.com/article/10.3390/bios13110984/s1. References [38,53,54,55,56,57,58,59,60,61,62,63,64] are cited in the Supplementary materials. Figure S1. Time-dependent growth process of 3D HWT arrays sample. Typical SEM images of the HWT grown under the different reaction time: (a) 15 min, (b) 30 min, (c) 60 min, (d) 90 min, (e) 120 min, and (f) 150 min. Figure S2. Full-range XPS spectra of the HWT-C sample. Figure S3. PL spectra of the HWT (red curve) and HWT-C (black curve) samples. Figure S4. Effect of pH value on photocurrent responses of sensor platform with the PSA concentration of 0.6 pg/mL in PBS buffer. Figure S5. Effect of incubation time of antigen with antibody on photocurrent responses of sensor with the PSA concentration of 0.6 pg/mL in PBS buffer (0.01 mol/L, pH 7.4). Table S1. Decay parameters and average lifetime according to a bi-exponential fitting model of the PL decay curves obtained from the samples. Table S2. Comparison of previously reports methods for the detection of PSA. Table S3. Determination of PSA in human serum samples. Table S4. Comparison of previously other materials for the detection of PSA.
