# Supplementary material for: Insights into Chemical Bonds for Eliminating the Depletion Region and Accelerating the Photo-Induced Charge Efficient Separation toward Ultrasensitive Photoelectrochemical Sensing
Source: Biosensors (Basel). 2023 Nov 13;13(11):984. doi: 10.3390/bios13110984 (PMC10668988; doi:10.3390/bios13110984)
Supplement: Supplementary file 1 [file biosensors-13-00984-s001.zip › biosensors-2560366-supplementary.pdf]

Insights into Chemical Bonds for Eliminating the Depletion Region and Accelerating the Photo-Induced Charge Efficient Separation toward Ultrasensitive Photoelectrochemical Sensing

Shuai Wang [1], Haihan Yu [1], Shenguang Ge [2], Yanhu Wang [1,3,*], Chaomin Gao [1,*] and Jinghua Yu [1]

1 School of Chemistry and Chemical Engineering, University of Jinan, Jinan 250022, China

2 Institute for Advanced Interdisciplinary Research, University of Jinan, Jinan 250022, China

3 Key Laboratory for Applied Technology of Sophisticated Analytical Instruments of Shandong Province, Shandong Analysis and Test Center, Qilu University of Technology (Shandong Academy of Sciences), Jinan 250014, China

* Correspondence: wyhloving633@163.com (Y.W.); chm_gaocm@163.com (C.G.); Tel.: +86-0531-82767040 (C.G.)

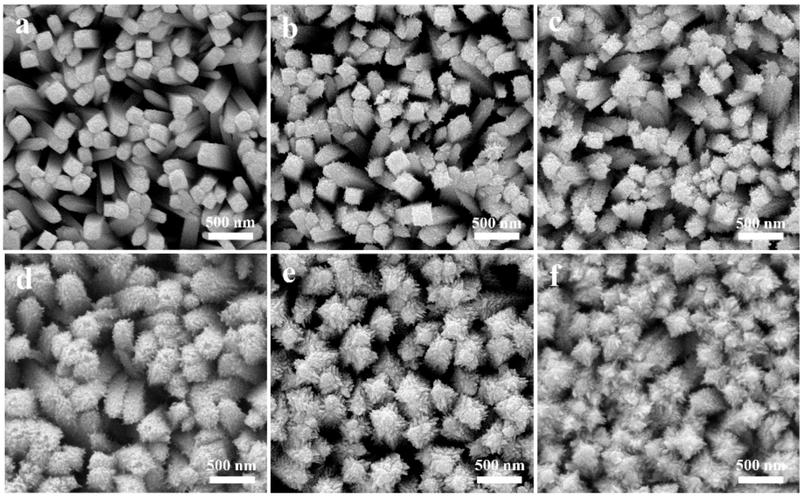

Figure S1. Time-dependent growth process of 3D HWT arrays sample. Typical SEM images of the HWT grown under the different reaction time: (a) 15 min, (b) 30 min, (c) 60 min, (d) 90 min 120 min, 150 min.

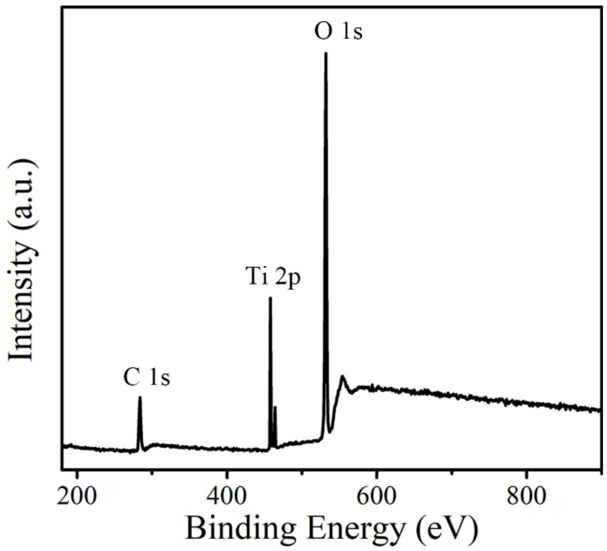

Figure S2. Full range XPS spectra of HWT-C sample.

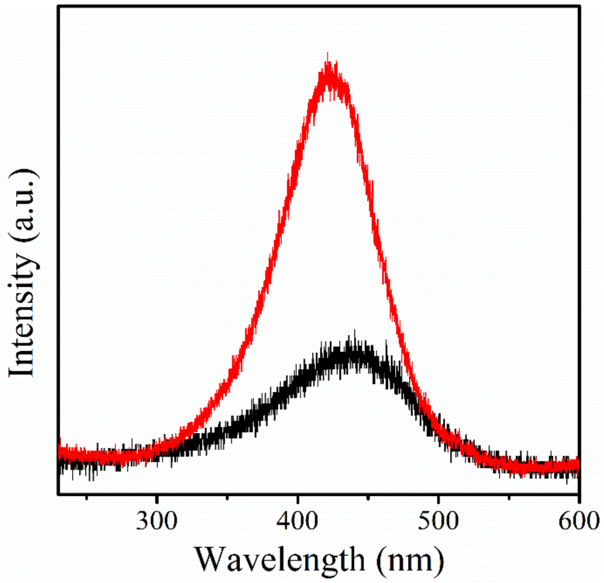

Figure S3. PL spectra of the HWT (red curve) and HWT-C (black curve) samples.

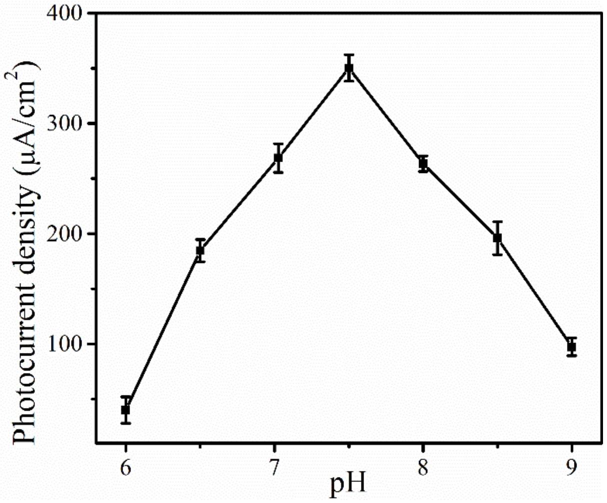

Figure S4. Effect of pH value on photocurrent responses of sensor platform with the PSA concentration of 0.6 pg/mL in PBS buffer.

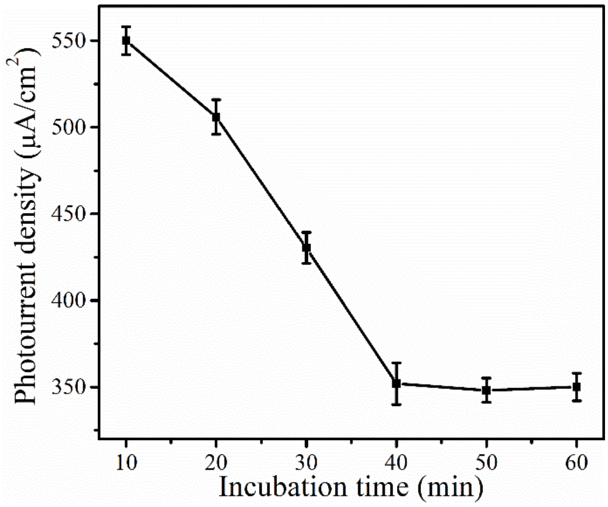

Figure S5. Effect of incubation time of antigen with antibody on photocurrent responses of sensor with the PSA concentration of 0.6 pg/mL in PBS buffer (0.01 mol/L, pH 7.4).

Table S1. Decay parameters and average lifetime according to a bi-exponential fitting model of the PL decay curves obtained from the samples.

Samples	τ_1 (ns)	τ_2 (ns)	A_1	A_2	τ_{ave} (ns)
HWT	9.25	6.78	2.3	1.19	8.57
HWT-C	4.33	3.68	4.6	2.12	4.14

Table S2. Comparison of previously reports methods for the detection of PSA.

Methods	Linear range	Detection limit	References
Differential pulse voltammetry	2 ng/mL-80 ng/mL	1 pg/mL	[2]
Electrochemiluminescence	1 pg/mL-10 ng/mL	0.72 pg/mL	[3]
Immunochromatography	0.5 pg/mL-200 pg/mL	2.05 pg/mL	[4]
Biofuel cell	0.3 pg/mL-7 ng/mL	0.1 pg/mL	[5]
Differential pulse voltammetry	1 pg/mL-30 ng/mL	0.78 pg/mL	[6]
PEC	0.02 pg/mL-100 ng/mL	0.007 pg/mL	This work

Table S3. Determination of PSA in human serum samples.

Number	Content of PSA (ng/mL)	Detection	Recovery (%)
1	0.02	0.021	105
2	0.1	0.098	98.0
3	1.0	0.96	96
4	10.0	10.2	102
5	50.0	49.3	98.6
6	100.0	98.5	98.5

Table S4. Comparison of previously other materials for the detection of PSA.

Materials	Linear range	Detection limit	References
Pt SA-$Zn_{0.5}Cd_{0.5}S$	0.001 ng/mL-10 ng/mL	0.22 pg/mL	[7]
PI5CA/WO_3	0.5 pg/mL-50 ng/mL	0.12 pg/mL	[8]
CdS nanorods	0.005 ng/mL-50 ng/mL	0.0018 ng/mL	[9]
PDANP	0. 05 pg/mL-50 ng/mL	0.027 pg/mL	[10]
rGO-$BiFeO_3$	10 pg/mL-100 ng/mL	0.3 pg/mL	[11]
Ag_2S/CuS/α-Fe_2O_3	0.01 pg/mL-10ng/mL	0.0033 pg/mL	[12]
ERGO-TiO_2	0.02 pg/mL-200 ng/mL	0.0068 pg/mL	[13]
3D HWT-C	0.02 pg/mL-100 ng/mL	0.007 pg/mL	This work

References:

1. Gao, C.; Wei, T.; Zhang, Y.; Song, X.; Huan, Y.; Liu, H.; Zhao, M.; Yu, J.; Chen, X. A photoresponsive rutile TiO_2 heterojunction with enhanced electron-hole separation for high-performance hydrogen evolution. *Adv. Mater.* **2019**, *31*, 1806596.
2. Zhao, M.; Fan, G. C.; Chen, J. J.; Shi, J. J.; Zhu, J. J. Highly sensitive and selective photoelectrochemical biosensor for Hg^{2+} detection based on dual signal amplification by exciton energy transfer coupled with sensitization effect. *Anal. Chem*. **2015**, *87*, 12340-12347.
3. Kavosi, B.; Salimi, A.; Hallaj, R.; Amani, K. A highly sensitive prostate-specific antigen immunosensor based on gold nanoparticles/PAMAM dendrimer loaded on MWCNTS/chitosan/ionic liquid nanocomposite. *Biosens. Bioelectron.* **2014**, *52*, 20-28.
4. Zhang, N.; Gao, H.; Xu, C. H.; Cheng, Y.; Chen, H. Y.; Xu, J. J. An Efficient Electrochemiluminescence Enhancement Strategy on Bipolar Electrode for Bioanalysis. *Anal. Chem.* **2019**, *91*, 12553-12559.
5. Fang, C. C. C., C. C. Yang, Y. Q. Wei-Kai, T. Wang, Y. T. Chan, Y. H. Multiplexed Detection of Tumor Markers with Multicolor Polymer Dot-Based Immunochromatography Test Strip. *Anal. Chem.* **2018**, *90*, 2134-2140.
6. Gao, C.; Zhang, L.; Wang, Y.; Yu, J.; Song, X. Visible-light driven biofuel cell based on hierarchically branched titanium dioxide nanorods photoanode for tumor marker detection. *Biosens. Bioelectron.* **2016**, *83*, 327-333.
7. Li, B.; Guo, L.; Chen, M.; Guo, Y.; Ge, L.; Kwok, H. F. Single-atom Pt-anchored $Zn_{0.5}Cd_{0.5}S$ boosted photoelectrochemical immunoassay of prostate-specific antigen. *Biosens. Bioelectron.* **2022**, *202*, 114006.
8. Lu, Y.; Zhang, B.; Tian, Y.; Guo, Q.; Nie, G. Ultrasensitive ratiometric photoelectrochemical immunoassay for prostate specific antigen based on nanoscale heterojunction. Sensor. Actuat B: Chem. **2021**, *326*, 128994.
9. Zhang, K.; Lv, S.; Lin, Z.; Li, M.; Tang, D. Bio-bar-code-based photoelectrochemical immunoassay for sensitive detection of prostate-specific antigen using rolling circle amplification and enzymatic biocatalytic precipitation. *Biosens. Bioelectron.* **2018**, *101*, 159-166.

10. Yao, L.; Xu, J.; Shi, M.; Huang, Y.; Fang, L.; Zhao, S.; Chen, Z. F.; Liang, H. Polydopamine nanoparticle-based multicolor proximity immunoassays for ultrasensitive, multiplexed analysis of proteins using isothermal quadratic amplification. *Sensor. Actuat B: Chem*. **2019**, *282*, 626-635.
11. Zhou, Q.; Lin, Y.; Zhang, K.; Li, M.; Tang, D. Reduced graphene oxide/$BiFeO_3$ nanohybrids-based signal-on photoelectrochemical sensing system for prostate-specific antigen detection coupling with magnetic microfluidic device. *Biosens. Bioelectron.* **2018**, *101*, 146-152.
12. Chen, M.; Wang, C.; Meng, H.; Mo, F.; Fu, Y. A novel signal self-enhancement photoelec trochemical immunosensor without addition of a sacrificial agent in solution based on Ag_2S /CuS/α-Fe_2O_3 n-p-n heterostructure films. *Chem. Commun*. **2020**, *56*, 2300-2303.
13. Deng, K.; Wang, H.; Xiao, J.; Li, C.; Zhang, S.; Huang, H., Polydopamine nanospheres loaded with l-cysteine-coated cadmium sulfide quantum dots as photoelectrochemical signal amplifier for PSA detection. Anal. Chim. Acta **2019**, *1090*, 143-150.
